# Low complexity regions in the proteins of prokaryotes perform important functional roles and are highly conserved

**DOI:** 10.1093/nar/gkz730

**Published:** 2019-09-04

**Authors:** Chrysa Ntountoumi, Panayotis Vlastaridis, Dimitris Mossialos, Constantinos Stathopoulos, Ioannis Iliopoulos, Vasilios Promponas, Stephen G Oliver, Grigoris D Amoutzias

**Affiliations:** 1 Bioinformatics Laboratory, Department of Biochemistry and Biotechnology, University of Thessaly, 41500, Greece; 2 Microbial Biotechnology-Molecular Bacteriology-Virology Laboratory, Department of Biochemistry and Biotechnology, University of Thessaly, 41500, Greece; 3 Department of Biochemistry, School of Medicine, University of Patras, 26504, Greece; 4 Department of Medicine, University of Crete, Heraklion 71003, Greece; 5 Bioinformatics Research Laboratory, Department of Biological Sciences, New Campus, University of Cyprus, PO Box 20537, CY-1678 Nicosia, Cyprus; 6 Cambridge Systems Biology Centre & Department of Biochemistry, University of Cambridge, CB2 1GA, UK

## Abstract

We provide the first high-throughput analysis of the properties and functional role of Low Complexity Regions (LCRs) in more than 1500 prokaryotic and phage proteomes. We observe that, contrary to a widespread belief based on older and sparse data, LCRs actually have a significant, persistent and highly conserved presence and role in many and diverse prokaryotes. Their specific amino acid content is linked to proteins with certain molecular functions, such as the binding of RNA, DNA, metal-ions and polysaccharides. In addition, LCRs have been repeatedly identified in very ancient, and usually highly expressed proteins of the translation machinery. At last, based on the amino acid content enriched in certain categories, we have developed a neural network web server to identify LCRs and accurately predict whether they can bind nucleic acids, metal-ions or are involved in chaperone functions. An evaluation of the tool showed that it is highly accurate for eukaryotic proteins as well.

## INTRODUCTION

Many proteins contain segments of very low amino-acid diversity, termed Low Complexity Regions (LCRs) (1). LCRs were originally thought as the ‘junk’ part of a protein or as neutral linker regions between domains (2–4) and during bioinformatics analyses are usually filtered out when searching for homologs (5). However, emerging experimental evidence increasingly indicated that LCRs may play important adaptive and conserved roles that are highly relevant to biotechnology, heterologous protein expression, medicine, as well as to our understanding of protein evolution (6–9).

One of the established methodologies to identify LCRs is by measuring their Shannon entropy (1,10). The lower the value of the calculated entropy, the more homogeneous the region is in terms of amino acid content. Several computational tools have been developed to detect LCRs (11–17) (see especially (17) for a very recent and extended review on this topic). A subset of LCRs are single amino acid repeats (SARs) or tandem or interspersed repeats of short period (2–5 amino acids). Furthermore, repetition of large amino acid segments of >10 amino acids, or even motif or domain repeats are also categorized as protein repeats (9,18,19), but are not the subject of the current study.

The LCRs of eukaryotic proteins have been the focus of many past and recent studies, due to their involvement in human diseases (20), especially neurodegenerative ones. For example, hydrophobic LCRs tend to form amyloids in humans and other eukaryotes (21). From a mechanistic point of view, LCRs were originally proposed to be unstructured and flexible linkers that served to separate the structured (and functional) domains of complex proteins (3). However, LCRs are capable of forming secondary structures, like helices (more often) and even sheets (22). Individual investigations have often revealed that LCRs play a structural role in eukaryotic proteins such as collagens, myosin, keratins, silk, cell wall proteins (14), have adhesive roles (23), function in excreted sticky proteins used for prey capture (24), or have roles as transducers of molecular movement, e.g. in the prokaryotic TonB/TolA systems (25). The abundance of LCRs in antigenic proteins has been linked to antigen diversification and the rapid adaptation of microbial pathogens to the immune system (26). Depending on their amino acid content, LCRs may form surfaces for interaction with phospholipid bilayers (27), or as positive charge clusters for DNA binding (28,29), or as negative or even histidine-acidic charge clusters for coordinating calcium, magnesium or zinc ions (28). LCRs may also play important roles in protein translation, with significant consequences for recombinant protein production in biotechnology. They may function as tRNA ‘sponges’, slowing down translation in order to allow time for the correct folding of the nascent polypeptide chain (7). They may even function as frame-shift checkpoints, by shifting to an unusual amino acid content that makes the protein highly unstable or insoluble, which in turn triggers fast recycling, before any further cellular damage (30,31).

LCRs are also intriguing from a micro and macro evolutionary perspective. They may be generated by DNA slippage, recombination and repair (32). Thus, they are linked to recombination hotspots and may even possibly facilitate cross-over (26,33). By originating from genetic instability, they may cause, at the DNA level, a certain region of the protein to expand or contract and even cause frame-shifts (phase-variants) that affect microbial pathogenicity or provide raw material for evolution (34–36). Most intriguingly, they may provide a window into the very early evolution of life (37). During early evolution, when only few amino acids were available and the primary genetic code was still expanding its repertoire, the first proteins were assumed short, repetitive (38) and therefore, of low complexity (39). Thus, modern LCRs could represent primordial aspects of the evolution towards the protein world and may provide clues about the functions of the early proto-peptides.

Most studies have focused on the evolution, functional and structural role of LCRs, which are very prominent in eukaryotes. However, their role in prokaryotes has been overlooked, mostly because of the notion that are not as widespread or important (40,41). Some of the notable exceptions include a study in 42 prokaryotic species regarding the evolutionary pressures on simple sequence repeats within coding regions (42), whereas another study in 19 archaeal species analyzed simple sequence repeats (di- to penta-nucleotides) (43). However, a comprehensive study of prokaryotic LCRs from many diverse prokaryotic lineages could provide a unique opportunity to understanding the origin, evolution and nature of these regions. Due to the high effective population size and short generation times of prokaryotes, the *de novo* emergence of a mildly or moderately deleterious amino acid repeat or LCR should quickly be filtered out by strong selective forces. This must be especially the case for LCRs found in highly expressed proteins, since they should also have a great impact on the energy burden of protein translation (44,45), even more so in prokaryotes. Thus, any prokaryotic LCRs that constitute evolutionary accidents with no functional significance should not be fixed by genetic drift and consequently should not demonstrate any levels of conservation among moderately distant evolutionary relatives. On the contrary, any LCR found among homologs of several moderately distant prokaryotic species should very probably reserve a functional role.

The goal of this study is to utilize the abundance of publicly available sequence data and provide the first large-scale genome and proteome-level survey of LCRs in prokaryotes and phages to reveal: (i) how prominent they are, (ii) what properties they have, (iii) whether any particular amino acid content is related to particular molecular functions and (iv) to develop a computational tool that identifies such regions, finds other LCRs with similar amino acid content in other annotated proteins and predicts their functional role.

## MATERIALS AND METHODS

Archaeal, bacterial and bacteriophage proteomes were downloaded from the Uniprot/Swissprot FTP site (March/April 2017) (46). We retained only one representative proteome from each genus, thus resulting to 1334 bacterial, 102 archaeal and 102 bacteriophage proteomes. The goal was to enrich our datasets with conserved LCRs that would most probably have a functional role. From eukaryotes, the human, *Saccharomyces cerevisiae* and *Arabidopsis thaliana* proteomes were downloaded from Ensembl (47), *Schizosaccharomyces pombe* from Pombase (48) and *Drosophila melanogaster* from Flybase (49), where we kept the longest open reading frame from every protein coding gene.

We developed custom Perl scripts that calculated the Shannon entropy (10) of a given sliding amino acid sequence window within a protein. That sliding window had a size and step of 30 and 15 amino acids respectively. We also investigated an amino acid window size of 50, but we observed similar findings (results not shown). In order to set a statistically meaningful and very strict entropy threshold for LCRs, we performed permutation tests for each analyzed proteome. More specifically, for each proteome we calculated its background amino acid frequency and later shuffled each protein by using its amino acid length and that specific proteome's amino acid background frequency. This step was repeated 20 times. For each of these 20 shuffled proteomes from a given organism, we calculated the Shannon entropy of each 30 amino acid window. The lowest value of Shannon entropy obtained from all these 20 shuffled proteomes was used as an LCR threshold for that particular organism. Next, the Shannon entropy of every real protein fragment from that organism was calculated and only those that had a value below the estimated threshold (for that particular organism) were retained as LCRs. Overlapping protein fragments that passed the entropy threshold were merged into a single LCR. After this step, the retained LCRs were analyzed for their amino acid frequency and a corresponding vector for each LCR was calculated with the count for each amino acid (see Supplementary File 1). For the codon frequency analysis of LCRs, the DNA sequences (CDSs) of their proteins were retrieved from EMBL-Bank (50) by mapping Uniprot IDs to EMBL-bank IDs. Only protein coding DNA sequences that exactly matched the Uniprot protein sequences were retained. Codon frequency was estimated for each LCR with a custom Perl script. Single amino acid repeats were identified by a custom made Perl script that analyzed the LCRs for stretches of the same amino acid with a length of 10 or above.

LCRs were clustered, based on the absolute count of amino acids within each LCRs. Amino acids with a count < 3 were filtered out and the resulting filtered vectors were clustered and visualized with the clustergram function in Matlab.

In order to identify the functional categories of the proteins that contained LCRs, their Gene Ontologies (51) were retrieved from Uniprot.

For phylogenetic analyses of homologous proteins that contained LCRs, the Muscle (52), Seaview v4 (53) and Jalview (54) software packages were used for generating and visualizing multiple alignments and phylogenetic trees (BioNJ method). Visualization and marking of the LCRs within the published protein 3D structures was performed with the PyMol software (55).

Keras/Tensorflow was used to build and train the Neural Networks (NN) using, as parameters, one hidden layer of 10 nodes, a batch size of 60, 152 epochs of training and a dropout value of 0.16, (loss: categorical crossentropy; optimizer: adam; activation: relu; all other parameters default). A web server, named LCR hound was developed based on the Jhipster Application Framework, which utilizes the Java language and Spring Framework for the back-end and Angular Javascript Framework for the front-end. LCR hound is freely available at: http://bioinf.bio.uth.gr/lcr/.

## RESULTS AND DISCUSSION

### Prevalence of LCRs in prokaryotes

In 98.7% (1316/1334) of the analyzed bacterial proteomes, we identified 22 259 LCRs in 20 788 proteins. In 100% (102/102) of the analyzed archaeal proteomes we identified 1521 LCRs in 1459 proteins. In 50% (51/102) of the analyzed phage proteomes, we identified 116 LCRs in 106 proteins. On average, 0.05 and 0.07% of the bacterial and archaeal proteomes (total amino acids of LCRs in a given proteome/total amino acids of that proteome) were forming LCRs (based on our very stringent criteria) whereas for the five eukaryotic proteomes (human, fruitfly, yeast, fission yeast, *Arabidopsis*) this coverage was significantly higher (on average, 0.4%; between 2 and 23 times higher than prokaryotes). We thus verify previous observations that LCRs are more abundant in eukaryotes, but they also have a significant presence in many prokaryotes. Of note, LCR-hound identified 892 280 amino acids being within LCRs. Of those, 99.7% were also identified by SEG being within LCRs (based on Shannon entropy), or by fLPs (56) (being within compositionally biased regions), or both of them (90.3%) (see Supplementary File 1, worksheet ‘Overlap_with_other_software’).

The average size of a bacterial, archaeal and phage LCR was 38, 36 and 33 amino acids respectively. In bacteria, the largest LCR was 1110 amino acids long (40% of the protein), in the hemagglutinin (Uniprot ID: F9Q6P8) of *Haemophilus pittmaniae*. In archaea, the largest LCR was 390 amino acids long (52% of the protein) in an uncharacterized protein (Uniprot ID: Q0W1C5) of *Methanocella arvoryzae*. In phages, the largest LCR was 115 amino acids long (46% of the protein) in the Gp39 (Uniprot ID: Q3V5F6) of *Corynebacterium phage BFK20*. Our analysis of the five Eukaryotes (human, fruitfly, yeast, fission yeast, *Arabidopsis*) showed that the average size of an LCR was 42 amino acids long, whereas the longest one was 960 amino acids long (75% of the protein) in the Sgs1 protein (Flybase ID: FBpp0077084) of *D. melanogaster*. Thus, eukaryotic LCRs tend to be longer than prokaryotic LCRs.

In the Archaea, the halobacterium *Natrialba magadii* has the highest number of LCRs and the highest enrichment for LCRs. They were found in many diverse categories of proteins, but a significant number of them were found in 41 proteins of unknown function. It has been argued that many proteins of the *Natrialba* genus have been adapted for the high salinity within the cell, by increasing the content of acidic amino acids on the surface of proteins (57). These extra acidic amino acids are thought to compete with ions for water and thus help increase the solubility of the proteins (58). Accordingly, we observed that 39% (1802/4607) of the amino acids of LCRs in this organism are indeed acidic (D or E) whereas the expected background frequency of acidic amino acids in LCRs in Archaea is 23% (12524/54154), an enrichment that is statistically significant (hypergeometric test *P*-value < 1e-142). Moreover, we found that the other 22 analyzed halobacteria also had (on average) 2.3 times more acidic amino acids in their LCRs than the LCRs of the other 78 non-halophilic Archaea (34 versus 15% acidic amino acids in LCRs, respectively,—5941/17478 versus 4781/32069; hypergeometric test *P*-value 0). Thus, it is likely that these much more acidic LCRs in *Natrialba* and in halobacteria in general have a role in the adaptation of the cellular machinery in halophilic environments.

In bacteria, the organism with the highest enrichment for LCRs is *Enhygromyxa salina*, a delta proteobacterium that belongs to myxobacteria. Intriguingly, four of the top five bacteria with the highest enrichment for LCRs are also myxobacteria. This lineage is of particular interest because they have very large genomes, produce many biotechnologically important metabolites, and behave like transitory multicellular aggregates under stress conditions, where cells from the same aggregate form patterned multicellular structures (59). It is conceivable that such a high number of LCRs is related to the unusual features and multicellular aggregates of this taxonomic group, but further investigation is needed. LCRs from this lineage were found in many diverse categories of proteins, but the majority (52%) of LCRs were found in proteins of unknown function. Intriguingly, glycine was the most frequent amino acid (25%) in LCRs of this lineage, whereas the average background frequency of glycine in bacterial LCRs is 17%. In addition, aspartate was the second most frequent amino acid (15%) in LCRs of this lineage, whereas the average background frequency of aspartate in bacterial LCRs is 6%.

### Amino acid content of LCRs in prokaryotes

**Figure 1.**
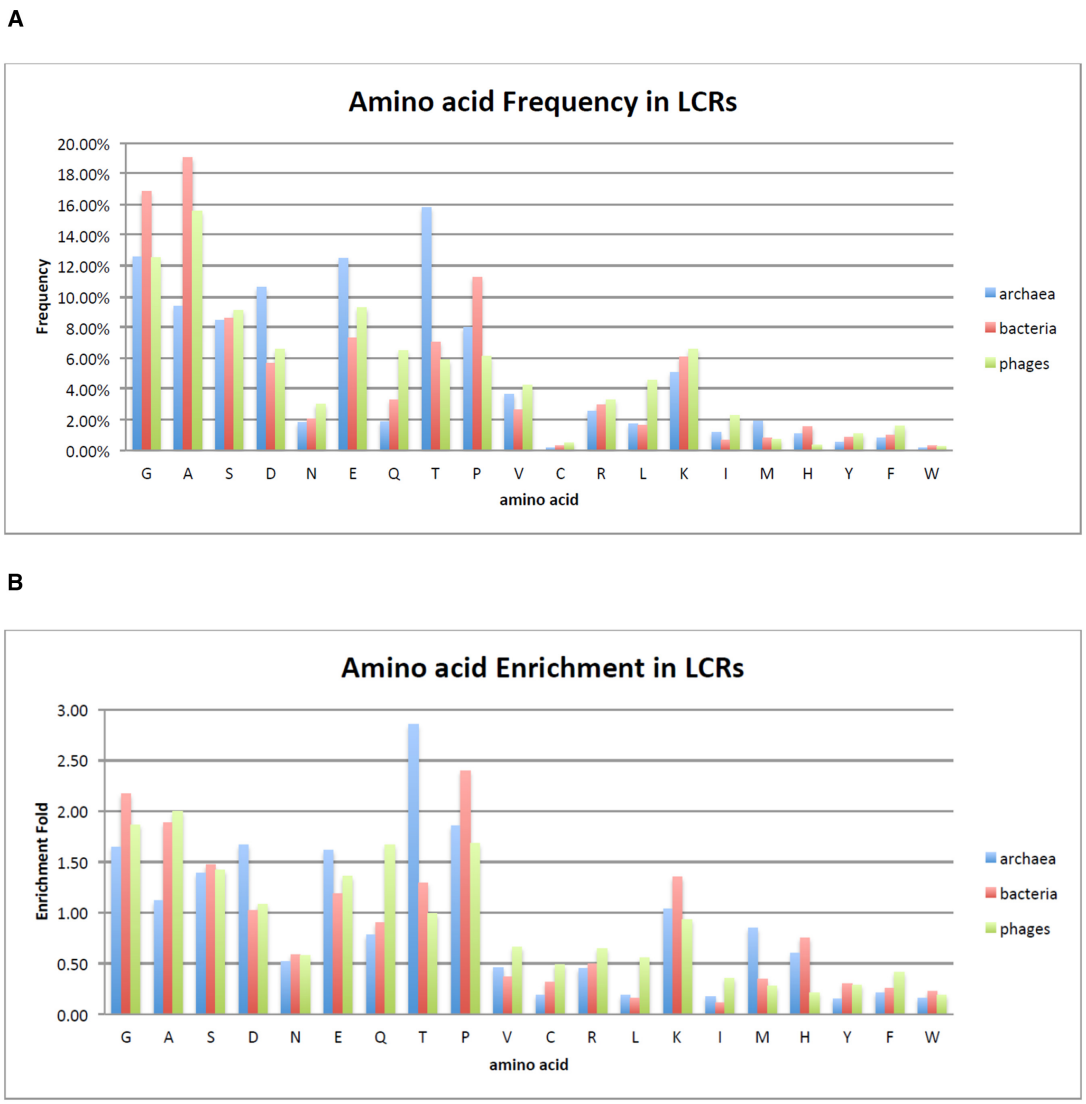
(**A**) Frequency and (**B**) enrichment of amino acids in LCRs. Enrichment was based on the background frequency obtained from the complete set of analyzed proteomes. The order of amino acids in the graphs is based on their biosynthetic energetic cost, as calculated in (44).

The frequency of each amino acid in the identified LCRs was calculated and compared to its background frequency in the complete set of analyzed proteomes in order to estimate its enrichment. Figure 1 summarizes the frequency (A) and enrichment (B) of amino acids in LCRs of Bacteria, Archaea and Phages.

Figure 1 shows that the three most enriched amino acids within LCRs of Bacteria are proline, glycine and alanine, whereas in Archaea they are threonine, aspartate and proline. In Phages, they are alanine, glycine and proline. Glycine and proline emerge as very enriched amino acids in all three evolutionary lineages, whereas alanine is highly enriched in Bacteria and Phages but not enriched in Archaea. On the other hand, hydrophobic (M, I, L, V) and aromatic amino acids (F, Y, W) as well as cysteine, arginine and asparagine are heavily under-represented in LCRs. All results of amino acid frequency and enrichment within LCRs for each of the three major lineages is shown in Supplementary File 1. Very similar trends for amino acids with a high (G, A, P, S, Q) and low (M, V, L, I, W, F, R, C) occurrence within LCRs have been observed in eukaryotes as well (8,40). Intriguingly, tandem repeats of short oligopeptides that are rich in glycine, proline, serine or threonine are capable of forming flexible structures that bind ligands under certain pH and temperature conditions (60). In addition, several studies highlight the structural role of proline-rich regions (25,61). Proline is a well-know alpha-helix breaker, however, amino acid repeats comprised of proline may form poly-proline helices (62).

This observed pattern of certain amino acids being over-represented (enriched for) or under-represented in LCRs could be partially explained by the energy cost for synthesis or metabolism of each of the amino acids. The aromatic amino acids (WFY) exhibit the highest energy cost, the hydrophobic amino acids (MILV) have a moderate cost, whereas glycine, alanine and serine have the lowest cost (44). Of note, these three amino acids also have small side chains and allow for more flexibility. Bioinformatics and evolutionary analyses have shown a preference for the use of amino acids with low energy cost in highly expressed bacterial proteins (44). Furthermore, Systems Biology approaches in the eukaryotic model *S. cerevisiae* (yeast) have also shown that amino acids with high energy cost are only used when they play a particular functional or structural role within a protein (45). We observed in Bacteria that (i) the Pearson correlation coefficient between background amino acid frequency and the energy cost of amino acid is −0.529, (ii) the Pearson correlation coefficient between LCR amino acid frequency and the energy cost of amino acid is −0.584 and (iii) the Pearson correlation coefficient between LCR amino acid enrichment and the energy cost of amino acid is −0.579. Although for most amino acids within LCRs there is a moderately strong link between enrichment and energy cost, the energy cost theory does not explain the high enrichment of proline and lysine and the under-representation of asparagine.

Another possible explanation for this observed pattern of certain amino acids being over-represented (enriched for) or under-represented in LCRs (which does not exclude the previous explanation of energy cost) could be the reactivity of certain amino acids. Cysteine is a very reactive amino acid (63) that would not be tolerated in high numbers within a small region of a protein. Indeed, experiments of ectopic expression of long cysteine homopolymers in mammalian COS-7 cells resulted in low viability (64). Similarly, extremely hydrophobic regions can form non-specific protein–protein interactions among themselves and with other moderately hydrophobic regions (65,66) in mammalian cells. Thus, their presence may disturb the balance of protein-protein interaction networks within the cell, especially if the carrier proteins are highly expressed.

A third explanation may be based on micro-evolutionary forces and, more specifically, on the bias of DNA polymerase slippage for certain di- tri- or tetra-nucleotides. In such a case, we should observe a significant presence for certain codons in highly enriched amino acids. By analyzing the codon frequency of all identified LCRs, we found that GGC for glycine in Bacteria and Archaea and CCG for proline in Bacteria were significantly more prevalent than the other codons of the same amino acid (see Supplementary File 2 for frequencies of all amino acids).

The amino acids with the highest frequency in LCRs are glycine and alanine, with their respective codons GGC and GCC being the most frequent, as well as complementary. In eukaryotes and more specifically in chordates (such as human, mouse, chicken, zebrafish and sea squirt), alanine- and glycine-rich LCRs are over-represented in recently formed LCRs and probably are better tolerated by the cell (67). Intriguingly, it has also been suggested that they represent the very first two amino acids (68) and codons (39,69,70) of the early genetic code. Thus, these two codons and their respective amino acids must have been constituents of the earliest oligopeptides, with a length of 10–55 amino acids (71) and very low complexity. Based on several different criteria and sources of data, Higgs and Pudritz (68) suggest G, A, D, E, V, S, P, I, L, T as the early amino acids of the genetic code. Trifonov's work (39,69,70) largely agrees with this categorization and proposes that the early amino acids in chronological order are G, A, D, V, S, P, E, L, T, R. We observed that many of the amino acids of the suggested very early genetic code (with the exception of the hydrophobic ones) are significantly enriched in bacterial LCRs. Most of the later additions to the genetic code are significantly under-represented in bacterial LCRs. We hypothesize and propose that, in a cell-free environment, the early genetic code may have also produced low complexity oligo-peptides from valine and leucine. However, later on, within a more complex cellular environment, these highly hydrophobic LCRs became inappropriate or even toxic from a protein interaction perspective and have been selected against ever since. In addition, we further hypothesize that the very early protopeptides did not have a nucleic acid binding role. As we show in the section below (‘Amino acid enrichment for certain functional categories’ section), DNA and RNA-binding LCRs are highly enriched in glucine, arginine and lysine. However, arginine and lysine are not among the amino acids of the proposed early genetic code.

### LCR-containing homologs that appear in many organisms

Previous analyses on model and non-model eukaryotic proteomes have revealed that LCRs are frequently found in proteins involved in binding of nucleic acids (DNA or RNA), in transcription, receptor activity, development, reproduction and immunity whereas metabolic proteins are depleted of LCRs and SARs (3,8,72,73). By analyzing the Uniprot annotation of LCR containing proteins, we observed that 44% (9751/22259) of Bacterial and 44% (662/1521) of Archaeal LCRs are detected in proteins of unknown function (see Supplementary File 1). However, a significant number of proteins of known function (from many different species), especially those involved in translation and the ribosome, nucleic acid binding, metal-ion binding, and protein folding were also found to contain LCRs (see Table 1). The same conclusions were reached when analyzing the frequency of GO terms for proteins that contained LCRs. The GO term frequency results for all kingdoms are shown in Supplementary File 3.

**Table 1.** Bacterial and archaeal protein annotations (Uniprot) with the most LCRs

No. of LCRs	Uniprot annotation	Kingdom
321	Translation initiation factor IF-2	Bacteria
281	DNA topoisomerase 1	Bacteria
220	60 kDa chaperonin	Bacteria
208	Acetyltransferase component of pyruvate dehydrogenase complex	Bacteria
186	30S ribosomal protein S16	Bacteria
167	Dihydrolipoamide acetyltransferase component of pyruvate dehydrogenase complex	Bacteria
166	Protein TonB	Bacteria
152	Single-stranded DNA-binding protein	Bacteria
146	RNA-binding protein	Bacteria
135	Serine/threonine protein kinase	Bacteria
60	Thermosome	Archaea
40	50S ribosomal protein L12	Archaea
23	Extracellular solute-binding protein family 5	Archaea
18	Chaperone protein DnaK	Archaea
13	50S ribosomal protein L10	Archaea
13	30S ribosomal protein S24e	Archaea
11	Prefoldin subunit alpha	Archaea
11	Carbohydrate binding family 6	Archaea
11	30S ribosomal protein S3	Archaea
7	Signal recognition particle receptor FtsY	Archaea

The first column shows the total number (sum) of LCRs that were found in all proteins (from many different organisms) with that particular Uniprot annotation.

It should be noted that we have also performed all of the above analyses and descriptive statistics for single amino acid repeats of size 10 or above, which constitutes a special subset of LCRs. The selection of a cut-off value of 10 was based on (71). All results are shown in Supplementary Files 4 and 5.

### LCRs are frequent in ribosomal proteins

Proteomics studies have shown that ribosomal proteins are among the most abundant (74) in the cell. We identified 822 and 117 LCRs from bacterial and archaeal ribosomal proteins respectively. On average, the most prominent amino acids in bacterial and archaeal ribosomal LCRs were alanine, glutamate and lysine (39, 21 and 9% in Bacteria and 27, 33 and 10% in Archaea, respectively,—see Supplementary File 6). About 49% (402/822) and 36% (42/117) of these bacterial and archaeal LCRs each had at least 70% of their amino acids as alanine and/or glutamate. 9% (77/822) and 15% (17/117) of these bacterial and archaeal LCRs each had at least 30% of their amino acids as lysine, thus strongly suggesting a role in nucleic acid binding. The bacterial proteins with more than 100 LCR-containing orthologs were S2, S16 and L25. Of note, each ortholog comes from a different genus. Furthermore, bacterial S3, S6, L3, L10, L17, L19, L21 and L31 had at least 20 LCR-containing orthologs each. In Archaea, S3, S24, L10 and L12 had at least 10 LCR containing orthologs each. By clustering the LCRs of each orthologous group, based on their amino acid content and counting their amino acid frequencies, we observed that the majority of bacterial LCRs of S16 (157/187), L3 (28/20), L10 (20/32), L19 (24/32) and of archaeal LCRs of S24 (7/13) and L12 (24/40) were mostly composed of alanine and glutamate (more than 70% of A and E amino acid content within LCRs). Notably the majority of bacterial L21 LCRs (34/45) had a high composition of lysine, more than 30%. From the 11 bacterial ribosomal proteins mentioned above, deletion studies in *Escherichia coli* have shown that S6, L21, L25 and L31 are non-essential, whereas the other seven are essential (75,76). Thus, the high prevalence of specific types of LCRs in so many and so abundant bacterial and archaeal ribosomal proteins that elevates the energy cost of ribosome biosynthesis strongly suggests that they must have a very important functional role that needs to be investigated further. Interestingly, ribosomal proteins have also been shown to have moonlighting functions beyond translation (77,78), thus it is conceivable that these LCRs could facilitate such functional innovations and adaptations. A computational analysis in the proteome of the model eukaryote *S. cerevisiae* revealed that terminally located LCRs have different biological roles from centrally located LCRs (79). More specifically, proteins with LCRs located at the N- or C- terminus have more protein-protein interactions and many of them are involved in protein translation. Intriguingly, we observed that the vast majority of the bacterial and archaeal ribosomal LCRs were located at the C-terminus of their proteins (see Figure 16 within Supplementary File 7). By studying the available crystal structures of the ribosome we observed that these LCRs should also be located at the surface of the ribosome (see Supplementary File 7). Thus, they could potentially be involved in novel molecular interactions within or outside the framework of translation. It is conceivable that at an early phase of the evolution of the ribosome, ancient proteins that were once at its surface also contained LCRs, or even evolved from LCRs. However, as the ribosome further evolved and new proteins were added (80), the ancient proteins became buried within its core, had their sequences optimized for a certain function and were ‘frozen’ (in evolutionary terms). Thus, at this later stage, they were selected against containing highly unstable LCRs.

### Amino acid enrichment for certain functional categories

For every GO term that contained at least 50 LCRs, we calculated the frequency of every amino acid in the corresponding LCRs and the fold-enrichment, compared to the background frequency. As a background frequency for a certain amino acid in a certain kingdom, we used all the LCRs identified in Bacteria, Archaea and Phages. We further focused on those notable cases where the fold-enrichment was ≥2.5 times higher than the background (see Supplementary File 8). We observed five major trends. The first is that proteins with GO terms related to polysaccharide binding and processing were enriched for serine and threonine in their LCRs (see Table 2). This observation is probably attributed to the high frequency of poly-serine tracts in this class of enzymes (see also Supplementary Files 4 and 5). However, a significant number of serine- and threonine-rich LCRs originated from only two organisms that have many genes that catabolize polysaccharides; thus, further investigation is needed.

**Table 2.** Enrichment fold for five amino acids within LCRs of bacterial proteins, related to polysaccharide binding and processing

	Bacterial LCRs					
GO description	GO ID	C	P	Q	S	T
chitin binding	GO:0008061				2.5	3.9
carbohydrate binding	GO:0030246				2.9	3.1
carbohydrate metabolic process	GO:0005975				3.2	2.8
hydrolase activity, hydrolyzing O-glycosyl compounds	GO:0004553				3.6	2.5
cellulose catabolic process	GO:0030245				4.2	2.5
cellulase activity	GO:0008810				4.2	
peptidoglycan binding	GO:0042834			2.7		
chitinase activity	GO:0004568		2.9			4.2
xylan catabolic process	GO:0045493	2.8			3.8	
cellulose binding	GO:0030248	3.8			6.8	
endo-1,4-beta-xylanase activity	GO:0031176	3.9			4.6	

Only enrichment folds ≥2.5 are displayed, for clarity.

The second notable trend is that proteins with GO terms related to RNA binding and processing were enriched for arginine in their LCRs (see Table 3). Indeed, biochemical studies in human and other vertebrate proteins have shown that intrinsically disordered regions rich in arginine as well as in glycine, forming the well-known RGG/RG motifs have been widely reported to be involved in RNA binding with degenerate specificity (81–87). Also, YGG motifs have been reported in many RNA-binding proteins as well (83,84). This is most probably attributed to interactions of the positively charged arginines with the RNA duplex–quadruplex junction, whereas neighboring glycines function as flexible hinges (88). However, disordered arginine-rich regions have been reported to be involved in DNA-binding as well (89).

**Table 3.** Enrichment fold for nine amino acids within LCRs of bacterial proteins, related to RNA binding and processing

GO description	GO ID	D	E	F	I	L	M	N	R	V
7S RNA binding	GO:0008312			2.8		3.9	22			
DNA-directed 5′-3′ RNA polymerase activity	GO:0003899	5.9	4.5	3.3	4.3	3.9				
polyribonucleotide nucleotidyltransferase activity	GO:0004654	2.9							10.4	
3′-5′-exoribonuclease activity	GO:0000175	2.97							10.4	
RNA processing	GO:0006396								7.5	
helicase activity	GO:0004386							4.3	6.8	
mRNA catabolic process	GO:0006402								5.9	
RNA binding	GO:0003723							2.5	4.9	
small ribosomal subunit	GO:0015935								3.2	
translation initiation factor activity	GO:0003743								2.9	
rRNA binding	GO:0019843								2.8	
endoribonuclease activity	GO:0004521								2.7	3.2
rRNA processing	GO:0006364								2.6	3
tRNA processing	GO:0008033								2.6	3.2
ribonuclease E activity	GO:0008995								2.5	3.6
translation	GO:0006412		2.8							
structural constituent of ribosome	GO:0003735		2.8							
ribosome	GO:0005840		3							
5S rRNA binding	GO:0008097		3.2							
transcription, DNA-templated	GO:0006351	3.5	2.5			2.5				

Only enrichment folds ≥2.5 are displayed, for clarity.

The third trend is that proteins with GO terms related to DNA binding and processing were especially enriched for lysine, but also for glycine, tyrosine, phenylalanine and glutamine in their LCRs (see Table 4). Indeed, lysine-rich positively charged LCRs are found in many eukaryotic and prokaryotic DNA-binding proteins such as histones, histone-like proteins, DNA topoisomerases, KU proteins and have been shown to be involved in non-sequence-specific DNA binding, probably due to electrostatic interactions with the negatively charged DNA (29,90–92). In addition, disordered poly-lysine peptides have also been reported to be involved in RNA-binding (83,86). On the other hand, glycine could play a supportive role as flexible hinge (88). Intriguingly, an analysis of crystal structures of protein domain–DNA complexes revealed that both lysine and arginine as well as tyrosine, phenylalanine and glutamine participate in protein-DNA interactions (93). Thus, we hypothesize that LCRs enriched for these specific amino acids may have evolved repeatedly to generate different optimized and ordered (from a structural point) DNA-binding domains.

**Table 4.** Enrichment fold for nine amino acids within LCRs of bacterial proteins, related to DNA binding and processing.

GO description	GO ID	F	G	H	K	L	N	P	Q	Y
DNA recombination	GO:0006310									5.1
DNA polymerase III complex	GO:0009360							2.6	2.5	
regulation of transcription, DNA-templated	GO:0006355						2.9			
DNA-templated transcription, initiation	GO:0006352					3.6				
DNA binding	GO:0003677				4.2					
chromosome condensation	GO:0030261				5.2					
chromosome	GO:0005694				5.8					
DNA topological change	GO:0006265				6.1					
DNA topoisomerase type I activity	GO:0003917				6.3					
nucleosome	GO:0000786				6.5					
nucleosome assembly	GO:0006334				6.5					
nucleotide binding	GO:0000166			3.1						
DNA repair	GO:0006281		2.6							4.8
single-stranded DNA binding	GO:0003697	2.6	3.2						3	5.4
DNA replication	GO:0006260	5.4	2.5						2.6	3.2
DNA-templated transcription, termination	GO:0006353						7.8		2.5	2.9

Only enrichment folds ≥ 2.5 are displayed, for clarity

The fourth notable observation is that proteins with GO terms related to metal binding and more specifically to cobalt or nickel-binding were enriched mostly for histidine but also for aspartate in their LCRs (see Table 5). Poly-histidine tags (of six or more consecutive H residues) are widely used for protein purification by binding to columns with nickel or cobalt, with micromolar affinity (94). Moreover, naturally occurring poly-histidine peptides found in the venom of the viper *Atheris squamigera* have been shown to bind Zn(2+), Ni(2+) and Cu(2+) and affect the function of venom metalloproteases (95). We further observed histidine-rich LCRs in some TonB-dependent receptors derived from gram-negative bacteria (Flavobacteria, γ-proteobacteria). TonB receptors in these types of Bacteria are associated with the uptake and transport of iron siderophore complexes and cobalamine (among others) (96,97). Next, we retrieved the 82 histidine-rich LCRs from proteins that were annotated by Uniprot to be involved in cobalamine biosynthesis and observed that they have an average length of 36 residues, of which 53% histidine, 23% aspartate, 9% glutamate (see Supplementary File 9). Structured domains with metal binding properties have very similar frequencies of amino acids that are involved in the coordination of the metal. For example, the three most frequent amino acids involved in cobalt coordination in structured domains are H, D and E (98). It is conceivable that these structured domains could have evolved from low-complexity regions of similar amino acid content as that of the metal coordinating amino acids. These naturally occurring nickel-cobalt-cobalamin-binding LCRs that we identified in this analysis may have been optimized by natural selection over millions of years and could be starting points for various biotechnological applications. For example, they could be exploited as high-affinity metal-binding tags in protein purification, as environmental biosensors or even as metal-sequestering regulators of other proteins that need certain metal ions (i.e. venom metalloproteases).

**Table 5.** Enrichment fold for amino acids within LCRs of bacterial proteins, related to protein folding and metal-ion binding

GO description	GO ID	D	F	G	H	I	K	M
Unfolded protein binding	GO:0051082		5.7	2.6	4			21.6
Protein refolding	GO:0042026			2.9				37.2
Protein folding	GO:0006457		7.4		6.3			
Heat shock protein binding	GO:0031072		21.5	3		5.2		
Metal ion binding	GO:0046872				4.9		3.1	
Zinc ion binding	GO:0008270		4.5					
Nickel cation binding	GO:0016151	2.7			33			
Metal ion transport	GO:0030001	5.3			21.6			
Cobalamin biosynthetic process	GO:0009236	2.7			29			

Only enrichment folds ≥ 2.5 are displayed, for clarity.

The fifth observation is that proteins with GO terms related to protein folding were enriched for glycine, methionine and phenylalanine in their LCRs (see Table 5). Indeed, GGM-rich tails in proteins such as GroEL (in Bacteria) and the Thermosome (in Archaea) form double-ring complexes (99). In particular, the GGM-rich LCRs in GroEL have been shown to assist in substrate protein encapsulation and be directly involved in protein unfolding (100). Within the Thermosome, they have been shown to affect the assembly and thermal stability of the complex (99). As further evidence of this highly conserved role, a GGF-rich LCR is required for proper function and is not merely a linker domain in the Sis1 protein, which is the DNAJ homolog in the model eukaryote *S. cerevisiae* (101).

### Development of a neural network web server for the identification and functional prediction of LCRs

Due to their nature, LCRs are highly dynamic in evolutionary terms and diversify their sequence in a very rapid manner. Consequently, an LCR that has been characterized functionally in a certain protein of a species may not be properly detected by classical sequence homology software in other homologs of other species. Thus, a computational tool is needed that can detect LCRs based on their Shannon entropy and predict their function based on amino acid content, instead of sequence homology.

We have developed LCR hound (freely available at: http://bioinf.bio.uth.gr/lcr/), a tool available via a web server that accepts proteins or entire proteomes in FASTA format and applies user-defined sliding windows and a Shannon entropy cut-off for identifying LCRs. The user may alternatively select to shuffle the proteome, in order to estimate the Shannon entropy cut-off. The webserver graphically displays each identified LCR on a protein, the Shannon entropy score, the coordinates and the sequence of the LCR as well as the two most prominent amino acids, together with their frequencies within that LCR. Furthermore, the server identifies LCRs from Uniprot-annotated prokaryotic sequences (which we have analyzed) that have the closest amino acid or bigram (di-amino acid) content (and not based on sequence similarity) with the identified LCR in the input sequence, using the Pearson correlation coefficient. This information is provided with the intent to give clues about the potential role of newly identified LCRs in sequences of unknown function. Finally, the tool uses a neural network that we developed to predict the function of the identified LCR, based again on its overall di-amino acid content.

In the previous section of this analysis, we observed that certain amino acids are highly enriched for LCRs in specific functional categories of proteins. Lysine and arginine are highly enriched in the LCRs of DNA- and RNA-binding proteins, histidine in LCRs of metal-binding proteins (more prominently for cobalt or nickel-binding) and glycine, methionine and phenylalanine in the LCRs of chaperones. We found that our observations and statistical analyses were corroborated by previously published experimental evidence. Given this validation, we created four sets of LCRs, using as a base the functional annotation of their proteins and we further filtered them by clustering of their amino acid content (see Supplementary File 10). All 591 lysine- and arginine-rich LCRs of Uniprot-annotated DNA- and RNA-binding proteins formed the ‘DNA-RNA-binding’ group. All 70 histidine-rich LCRs from Uniprot-annotated metal-binding proteins formed the ‘Metal-binding’ group. All 118 glycine/methionine/phenylalanine-rich LCRs from Uniprot-annotated chaperones formed the ‘Chaperones’ group. All 3753 LCRs from proteins that had other annotations formed the ‘Others’ group. Within the Others group, we further added glycine-rich LCRs from eukaryotic keratins and collagens, in order to increase the network's discrimination in eukaryotes. We did not create a separate category for polysaccharide-binding because most of these LCRs originated from only two species. Thus, it was not entirely clear to us whether this amino acid content was related to the actual function or it was related to poly-serine and poly-threonine tracts that may have affected genome instability, recombination and gene expansion of this category of proteins in these two particular species.

Next, 70% (randomly selected) of the LCRs of each of the four categories (DNA-RNA-binding, Metal-ion-binding, Chaperone, Other) formed the training dataset and the other 30% the evaluation dataset. Each LCR was encoded as a vector of 400 bigram frequencies. The training dataset was used to train a neural network with the Keras/Tensorflow, that would provide a score for each of the four categories. Based on the predictions of the evaluation dataset, we achieved an overall accuracy of 0.924 (see confusion matrix of Table 6). We also tested an LCR encoding of vectors of 20 amino acid frequencies, but the bigram encoding had a marginally better accuracy.

**Table 6.** Confusion Matrix of the neural network

	Actual Chaperones	Actual DNA or RNA binding	Actual Metal-ion binding	Actual Other	Precision
Predicted Chaperones	34	1	0	4	87.18%
Predicted DNA or RNA binding	0	120	0	39	75.47%
Predicted Metal-ion binding	0	0	20	1	95.24%
Predicted Other	0	57	1	1082	94.91%
Recall	100%	67.42%	95.24%	96.09%	

As an extra evaluation step, we also analyzed the two best-annotated eukaryotic proteomes, from human and yeast. In humans, 40/41 (97%) of our predicted DNA or RNA-binding LCRs were indeed found within the experimentally validated DNA or RNA-binding proteins, one predicted chaperone-LCR was found within the 60 KDa chaperone, whereas 5/10 metal-binding LCRs were found in calcium or heme or metal-ion binding proteins, based on their Uniprot annotation and/or GO ontology. In yeast, all 7 predicted DNA or RNA-binding LCRs were found within DNA/RNA binding proteins whereas the one predicted Chaperone-LCR was found within the mitochondrial HSP60. Thus, LCR-hound is a suitable tool for the prediction of the functions of LCR-containing proteins from prokaryotes and eukaryotes as well.

## CONCLUSION

This paper reports the first comprehensive large-scale analysis of LCR prevalence and properties in prokaryotes and phages. We applied very stringent filtering criteria for the detection of LCRs and have focused on regions that are conserved in many lineages, thus strongly filtering in favour of LCRs with a functional role in the cell. The analysis of more than 1500 prokaryotic and phage proteomes from diverse evolutionary lineages highlights the significant presence and important role of LCRs in these ancient lineages. The amino acid content and enrichment patterns of prokaryotic LCRs are similar to those observed in eukaryotic LCRs, thus strongly suggesting common mechanisms of emergence (of LCRs) and evolutionary pressures (on LCRs) throughout all kingdoms of life. More specifically, the biosynthetic energy cost and biochemical reactivity seem to be important for the formation and preservation of these regions. LCRs are also enriched for the amino acids of the early genetic code, thus providing a window into the possible functions of the early proto-peptides that, by definition, were of low complexity. Intriguingly, we observed an extremely high prevalence of LCRs in transitorily multicellular prokaryotes, the Myxobacteria. Concerning their functional role, many LCRs are repeatedly found in the translation machinery, the chaperones, in nucleic acid binding proteins, in metal-ion binding proteins and in polysaccharide binding and processing proteins. Furthermore, each category of LCRs is enriched in certain amino acids and this enrichment is supported by published experimental evidence. Finally, this enrichment profile has been utilized to develop a tool, based on a neural network and available on a web server, that identifies LCRs and predicts their functional role. The accuracy of the prediction tool is very high and is not restricted to prokaryotes, but also applies to eukaryotes from very diverse lineages such as vertebrates and fungi, thus supporting the view of the highly conserved functional nature of these particular regions. In the future, this knowledge may have great implications for such diverse fields as biotechnology, heterologous protein expression, expanded genetic codes, medicine and evolution.

## Supplementary Material

gkz730_Supplemental_Files
