# Supplementary material for: Low complexity regions in the proteins of prokaryotes perform important functional roles and are highly conserved
Source: Nucleic Acids Res. 2019 Sep 4;47(19):9998–10009. doi: 10.1093/nar/gkz730 (PMC6821194; doi:10.1093/nar/gkz730)
Supplement: gkz730_Supplemental_Files [file gkz730_supplemental_files.zip › SUPPFILE4_SARs_descr_stats.docx]

**Prevalence and amino acid content of SARs in Prokaryotes**

In order to investigate the prevalence of SARs, we defined them as homopolymers of at least 10 residues, based on experiments that model the prebiotic polymerization of amino acids (Ferris et al. 1996). We identified 1052 SARs in 856 bacterial proteins. We identified 107 SARs in 75 archaeal proteins. We identified 2 SARs in 2 phage proteins (see supplementary file 5).

The average size of a bacterial, archaeal and phage SAR was 16, 13, and 12 amino acids respectively (supplementary file 5). In Bacteria, the largest SAR was 256 amino acids long (poly-D; 46% of the protein), in an uncharacterized protein (Uniprot ID: M1PF17) of *Desulfocapsa sulfexigens*. In Archaea, the largest SAR was 36 amino acids long (poly-E; 9% of the protein) in an uncharacterized protein (Uniprot ID: R4W893) of *Salinarchaeum sp.* In phages, the largest SAR was 13 amino acids long (poly-S; 2% of the protein) in the Gp185 (Uniprot ID: Q0QZ40) of *Synechococcus phage syn9*. Table 1 shows the largest SAR for each amino acid. As a point of reference, in the 5 analyzed Eukaryotes, the average size of a SAR was 15 amino acids long, whereas the longest one was 61 amino acids long (poly-E; 75% of the protein) in the Replication factor C large subunit protein (TAIR ID: At5g03710) of *A. thaliana*. The second largest eukaryotic SAR was 58 amino acids long (poly-S; 2% of the protein) in the Trinucleotide repeat-containing gene 18 protein (Ensembl Vega ID: OTTHUMP00000200617.4) in humans.

| **Kingdom** | **AA of SARs** | **No. of repeats** | **No. of organisms** | **Dominant codon** | **Dominant codon % (second most frequent codon%)** | **Size of longest SAR for that aa** | **Organism with longest SAR for that aa** | **Protein with longest SAR for that aa** |
| --- | --- | --- | --- | --- | --- | --- | --- | --- |
| **Archaea** | **A** | 18 | 1 | GCT | 64.7 (26.9) | 13 | Methanocella arvoryzae | Uncharacterized protein |
| **Archaea** | **D** | 4 | 3 | GAC | 62 (38) | 27 | Natronococcus occultus | Uncharacterized protein |
| **Archaea** | **E** | 12 | 12 | GAA | 61.9 (38.1) | 36 | Salinarchaeum sp. | Uncharacterized protein |
| **Archaea** | **G** | 12 | 9 | GGC | 53 (28) | 21 | Haloarcula marismortui | Cell surface glycoprotein related protein |
| **Archaea** | **L** | 1 | 1 | CTT | 80 (20) | 10 | Methanoplanus limicola | Putative uncharacterized protein |
| **Archaea** | **P** | 1 | 1 | CCA/CCT | 33 (25) | 12 | Vulcanisaeta distributa | Uncharacterized protein |
| **Archaea** | **Q** | 1 | 1 | CAG | 58 (42) | 19 | Methanocella arvoryzae | Uncharacterized protein |
| **Archaea** | **R** | 1 | 1 | AGA | 52.6 (36.8) | 19 | Thermoplasmatales archaeon | Uncharacterized protein |
| **Archaea** | **S** | 8 | 6 | TCA | 24.1 (22.4) | 21 | Methanobrevibacter millerae | Uncharacterized protein |
| **Archaea** | **T** | 49 | 11 | ACT | 28.2 (26.1) | 32 | Staphylothermus marinus | Putative oligopeptide transport system substrate-binding protein |
| **Bacteria** | **A** | 51 | 44 | GCC | 29.1 (24.9) | 31 | Noviherbaspirillum sp. | Uncharacterized protein |
| **Bacteria** | **D** | 73 | 53 | GAC | 52 (48) | 256 | Desulfocapsa sulfexigens | Uncharacterized protein |
| **Bacteria** | **E** | 20 | 17 | GAA | 61 (39) | 26 | Haliangium ochraceum | Uncharacterized protein |
| **Bacteria** | **F** | 1 | 1 | TTC | 97 (3) | 30 | Bilophila wadsworthia | Uncharacterized protein |
| **Bacteria** | **G** | 260 | 197 | GGC | 50 (29) | 119 | Tepidimonas fonticaldi | Uncharacterized protein |
| **Bacteria** | **H** | 12 | 10 | CAT | 50.3 (49.7) | 22 | Acinetobacter guillouiae | Uncharacterized protein |
| **Bacteria** | **I** | 2 | 2 | ATT | 74 (22) | 13 | Berkelbacteria bacterium | Uncharacterized protein |
| **Bacteria** | **K** | 6 | 4 | AAG | 77 (23) | 18 | Bilophila wadsworthia | Uncharacterized protein |
| **Bacteria** | **L** | 1 | 1 | TTG | 100 (0) | 21 | Alcaligenes sp. | Uncharacterized protein |
| **Bacteria** | **N** | 20 | 16 | AAC | 59 (41) | 30 | Peptoanaerobacter stomatis | SLH domain protein |
| **Bacteria** | **P** | 136 | 104 | CCG | 47 (23) | 101 | Acidithiobacillus ferrivorans | Uncharacterized protein |
| **Bacteria** | **Q** | 20 | 19 | CAA | 52.2 (47.8) | 48 | Coxiellaceae bacterium | RNase III inhibitor |
| **Bacteria** | **S** | 368 | 102 | AGC | 24.9 (16.2) | 56 | Teredinibacter turnerae | Putative lipoprotein |
| **Bacteria** | **T** | 81 | 24 | ACG | 40 (38) | 25 | Ilumatobacter coccineus | Uncharacterized protein |
| **Bacteria** | **V** | 1 | 1 | GTT | 55 (27) | 11 | Bergeyella zoohelcum | Uncharacterized protein |
| Table 1. Summary statistics of SARs for each amino acid. | | | | | | | | |

The bacterium with the most SAR containing proteins (45 proteins, 136 SARs) was *Teredinibacter turnerae,* that belongs to gamma-proteobacteria. Accordingly, *Staphylothermus marinus,* was the Archaeon (Crenarchaeota) with the most SAR containing proteins (7 proteins, 12 SARs). In phages, both organisms *(Synechococcus phage syn9 & Phage phiJL001*) that were detected had 1 protein that contained 1 SAR. The top 10 Bacteria and Archaea with the highest number of proteins that contain SARs, along with the number of SARs for that organism are shown in Table 2. Intriguingly, *Teredinibacter turnerae* and *Saccharophagus degradans* are two organisms that carry many genes involved in degradation of terrestrial and aquatic polysaccharides (Weiner et al. 2008; Yang et al. 2009). The Serine SARs of these two organisms are found in many of their polysaccharide degrading enzymes. *Haliangium ochraceum, Chondromyces apiculatus* and *Sorangium cellulosum* that also have many SARs in their proteomes belong to myxobacteria.

Poly-Serine tracts were the most abundant in Bacteria, followed by poly-Glycine and poly-Proline tracts (see Table 1). In Archaea poly-Threonine tracts were the most abundant, followed by poly-Alanine, poly-Glycine and poly-Glutamate tracts (see Table 1). Very similar observations have been made for mammalian proteomes as well, where Poly-Alanine, poly-Glycine and poly-Proline tracts were also very frequent (Karlin et al. 2002; Albà and Guigó 2004). We did not detect any SARs (of minimum size of 10) for Cysteine, Methionine, Tyrosine and Tryptophan in any Bacteria or Archaea. Of note, these amino acids are also significantly under-represented in LCRs.

Since Poly-Serine tracts were the most abundant in Bacteria, we further investigated their properties. 42% (90/214 proteins - 55 different gene annotations) of proteins containing poly-Serine tracts are involved in polysaccharide/carbohydrate degradation and metabolism. In comparison, only 14% (117/856) of all SAR containing proteins are involved in polysaccharide/carbohydrate degradation and metabolism, a statistically significant difference (Hyper-geometric test p-value < 4e-38). Most of these poly-Serine containing proteins (90% - 198 proteins) derive from *Teredinibacter turnerae* and *Saccharophagus degradans*, the two microorganisms mentioned above that encode many polysaccharide degrading enzymes (Weiner et al. 2008; Yang et al. 2009). Most probably these repeats have a function in polysaccharide degradation. Alternatively, these repeats may cause a genetic instability that triggers massive gene expansion of this functional category of proteins, via non-homologous recombination (Verstrepen et al. 2005). However, in humans, poly-Serine tracts are detected in proteins involved in transcription regulation or DNA binding (Huntley and Golding 2006).

| **Bacteria** | | | **Archaea** | | |
| --- | --- | --- | --- | --- | --- |
| **Organism** | **No. of proteins** | **No. of SARs** | **Organism** | **No. of proteins** | **No. of SARs** |
| Teredinibacter turnerae | 45 | 136 | Staphylothermus marinus | 7 | 12 |
| Saccharophagus degradans | 40 | 88 | Thermogladius cellulolyticus | 6 | 7 |
| Ilumatobacter coccineus | 16 | 17 | Desulfurococcus kamchatkensis | 5 | 12 |
| Kutzneria sp. | 14 | 14 | Palaeococcus pacificus | 5 | 5 |
| Haliangium ochraceum | 13 | 14 | Natrialba magadii | 4 | 4 |
| Chondromyces apiculatus | 12 | 12 | Acidilobus saccharovorans | 3 | 5 |
| Saccharothrix espanaensis | 11 | 12 | Cenarchaeum symbiosum | 3 | 3 |
| Actinosynnema mirum | 9 | 11 | Haladaptatus sp. | 3 | 3 |
| Microbulbifer thermotolerans | 9 | 13 | Methanobrevibacter millerae | 3 | 3 |
| Sorangium cellulosum | 8 | 11 | Methanocella arvoryzae | 3 | 19 |
| Table 2. Top 10 Bacteria and Archaea with the highest number of SAR containing proteins. | | | | | |

In order to investigate whether there is a significant bias for certain codons in specific types of SARs, we searched for those types of SARs that satisfied two criteria: i) being present in at least 10 organisms and ii) the most common codon was at least 50% more frequent than the second most common codon. In both Archaea and Bacteria we found that the GAA codon, that encodes for Glutamic Acid, satisfies both criteria. Also, GGC that encodes for Glycine satisfies the second criterion in both Bacteria and Archaea, but does not satisfy the first criterion in Archaea, just marginally. In addition, CCG that encodes for Proline and AGC that encodes for Serine satisfy both criteria, but only in Bacteria. Thus, there is some evidence for codon bias in certain SARs, but we have not investigated background codon bias for these cases.

It has been suggested that one of the most frequent mechanisms of LCR formation is expansion of oligonucleotide repeats by DNA-slippage that will lead to formation of amino acid repeats (Levinson and Gutman 1987; Chakraborty et al. 1997; Huntley and Golding 2006). Studies in vertebrates have shown that the emergent repeats will either degenerate due to point mutations or will be preserved by purifying selection (Radó-Trilla and Albà 2012). We further investigated to what extent the various types of prokaryotic SARs were predominantly composed of a single codon or had a mix of codons instead. Towards this goal, we applied two stringent criteria i) we investigated types of SARs in Bacteria and Archaea that had 20 or more instances in our datasets ii) we used an arbitrary but reasonable cutoff of a minimum prevalence of 80% for the most frequent codon within a particular SAR. This 80% cutoff allows for a small number of synonymous point mutations within the SAR. In Bacteria, less than 25% of SARs fulfilled the above criteria in poly-A, poly-D, poly-E, poly-G, poly-P, poly-Q, poly-S and poly-T tracts, whereas in Archaea, none of the poly-T SARs fulfilled them. A notable exception was poly-N in Bacteria, where 13/20 SARs (65%) fulfilled the criteria. An interpretation of the above findings is that most of these SARs are quite old and have accumulated many synonymous point mutations. Otherwise, a very recent SAR that emerged by DNA polymerase slippage would be composed of a single codon. An alternative explanation is that the SARs that do not fulfill the above criteria are not tri-nucleotide expansions, but other poly-nucleotide expansions (e.g. hexanucleotides). However, manual inspection revealed that only a very small number of these SARs are composed of two codons with a frequency of 50% each. Thus, this particular analysis strongly hints that most of the SARs are rather old, in evolutionary terms.

**Literature**

Albà MM, Guigó R. 2004. Comparative analysis of amino acid repeats in rodents and humans. Genome Res. 14:549–554.

Chakraborty R, Kimmel M, Stivers DN, Davison LJ, Deka R. 1997. Relative mutation rates at di-, tri-, and tetranucleotide microsatellite loci. Proc. Natl. Acad. Sci. U.S.A. 94:1041–1046.

Ferris JP, Hill AR, Liu R, Orgel LE. 1996. Synthesis of long prebiotic oligomers on mineral surfaces. Nature 381:59–61.

Huntley MA, Golding GB. 2006. Selection and slippage creating serine homopolymers. Mol. Biol. Evol. 23:2017–2025.

Karlin S, Brocchieri L, Bergman A, Mrazek J, Gentles AJ. 2002. Amino acid runs in eukaryotic proteomes and disease associations. Proc. Natl. Acad. Sci. U.S.A. 99:333–338.

Levinson G, Gutman GA. 1987. Slipped-strand mispairing: a major mechanism for DNA sequence evolution. Mol. Biol. Evol. 4:203–221.

Radó-Trilla N, Albà M. 2012. Dissecting the role of low-complexity regions in the evolution of vertebrate proteins. BMC Evol. Biol. 12:155.

Verstrepen KJ, Jansen A, Lewitter F, Fink GR. 2005. Intragenic tandem repeats generate functional variability. Nat. Genet. 37:986–990.

Weiner RM, Taylor LE, Henrissat B, Hauser L, Land M, Coutinho PM, Rancurel C, Saunders EH, Longmire AG, Zhang H, et al. 2008. Complete genome sequence of the complex carbohydrate-degrading marine bacterium, Saccharophagus degradans strain 2-40 T. PLoS Genet. 4:e1000087.

Yang JC, Madupu R, Durkin AS, Ekborg NA, Pedamallu CS, Hostetler JB, Radune D, Toms BS, Henrissat B, Coutinho PM, et al. 2009. The complete genome of Teredinibacter turnerae T7901: an intracellular endosymbiont of marine wood-boring bivalves (shipworms). PLoS ONE 4:e6085.
