# Supplementary material for: Low complexity regions in the proteins of prokaryotes perform important functional roles and are highly conserved
Source: Nucleic Acids Res. 2019 Sep 4;47(19):9998–10009. doi: 10.1093/nar/gkz730 (PMC6821194; doi:10.1093/nar/gkz730)
Supplement: gkz730_Supplemental_Files [file gkz730_supplemental_files.zip › SUPPFILE7_LCR_ribosome_images_R.docx]

**Crystal structures of the Bacterial and Archaeal Ribosomes.**

We used PyMOL to visualize the 3D structure of bacterial and archaeal ribosomes (see figures 1-15). For that purpose, the PDB entries for 50S(3I8I) and 30S(3I8H) ribosomal subunits of *Thermus Thermophilus* and 50S(3J21), 30S(3J20) ribosomal subunits and 50S ribosomal RNA(3J2L) of *Pyrococcus furiosus*(protein L12 from *Pyrococcus horikoshii*, 3WY9) were downloaded from RCSB PDB. The ribosomal proteins that we studied were colored blue, ribosomal RNA of both subunits was colored orange, tRNAs were colored green and mRNA was colored pink. In the images of the ribosome below, each protein along with the C-terminal residues (colored red) are shown as spheres. Since the vast majority of ribosomal LCRs are found at the C-terminus of their protein (see figure 16), the goal was to investigate whether the added C-terminal LCRs would be found at the surface of the ribosome, or buried. In the vast majority of proteins, the C-terminus was at the surface and could potentially make interactions with other molecules.

| 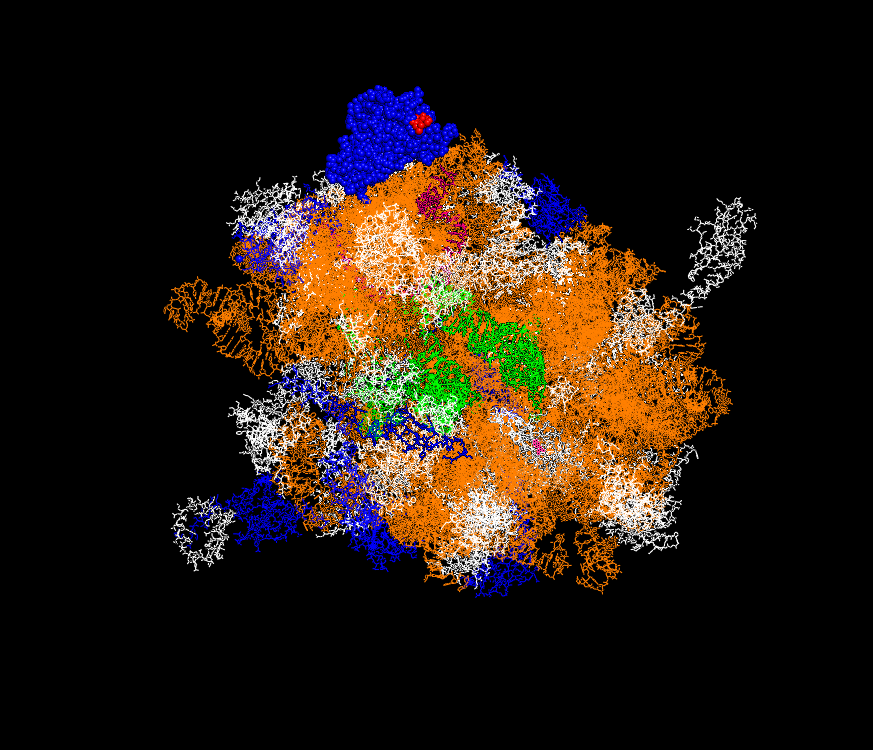 |
| --- |
| Figure 1. Crystal structure of the bacterial ribosomal S2 protein together with other ribosomal proteins and RNAs. |

| 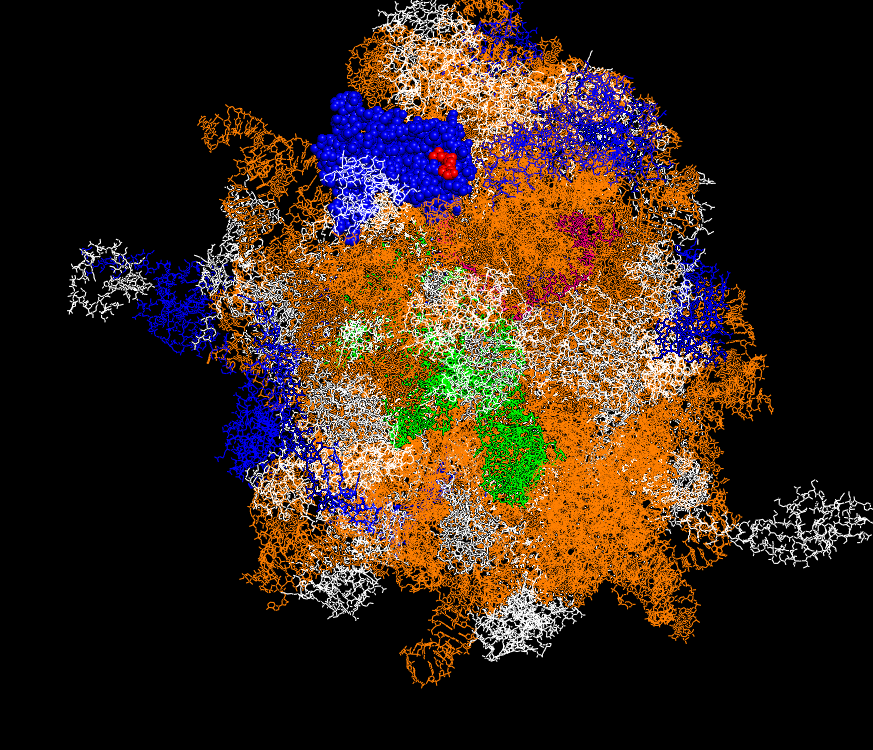 |
| --- |
| Figure 2. Crystal structure of the bacterial ribosomal S3 protein together with other ribosomal proteins and RNAs. |

| 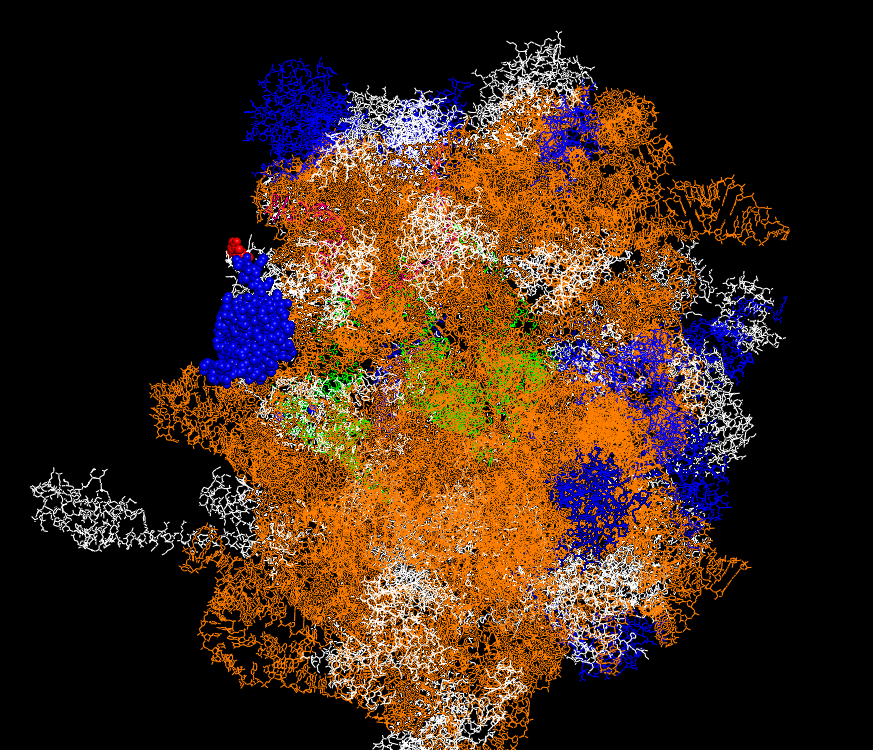 |
| --- |
| Figure 3. Crystal structure of the bacterial ribosomal S6 protein together with other ribosomal proteins and RNAs. |

| 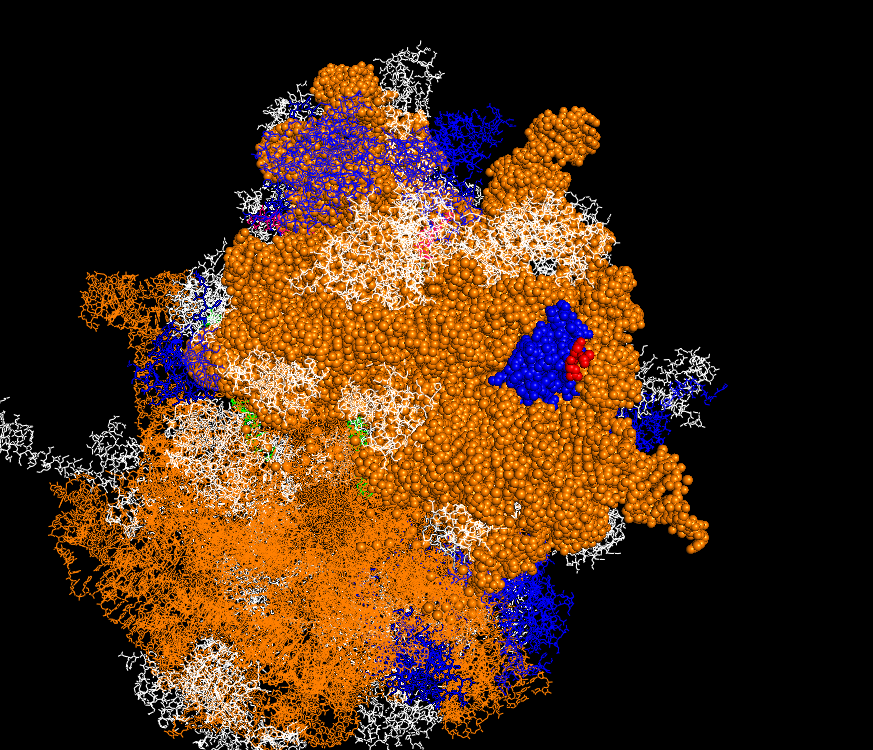 |
| --- |
| Figure 4. Crystal structure of the bacterial ribosomal S16 protein together with other ribosomal proteins and RNAs. |

| 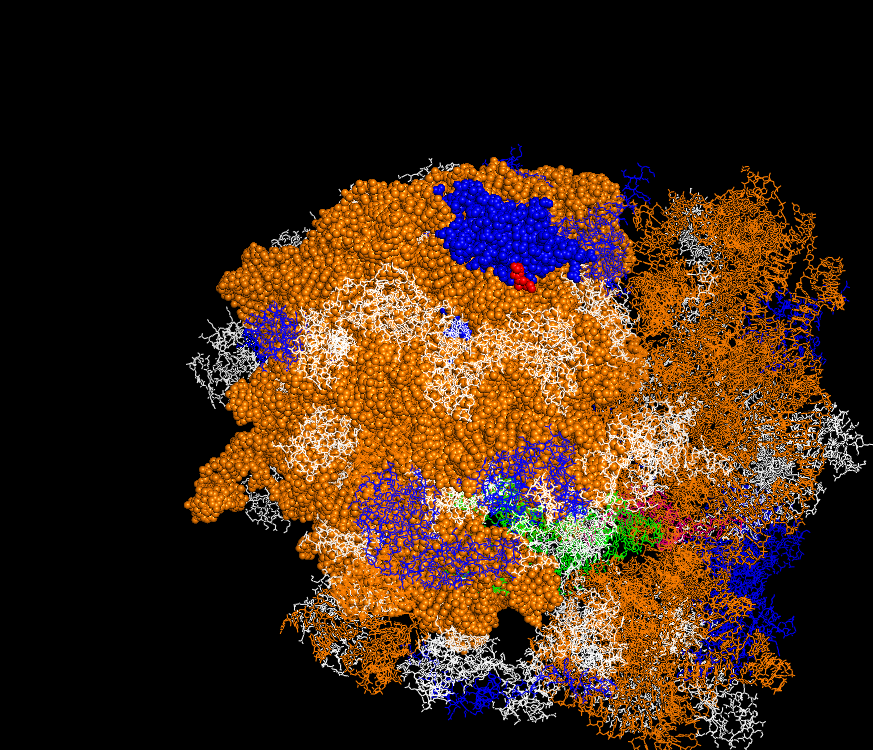 |
| --- |
| Figure 5. Crystal structure of the bacterial ribosomal L3 protein together with other ribosomal proteins and RNAs. |

| 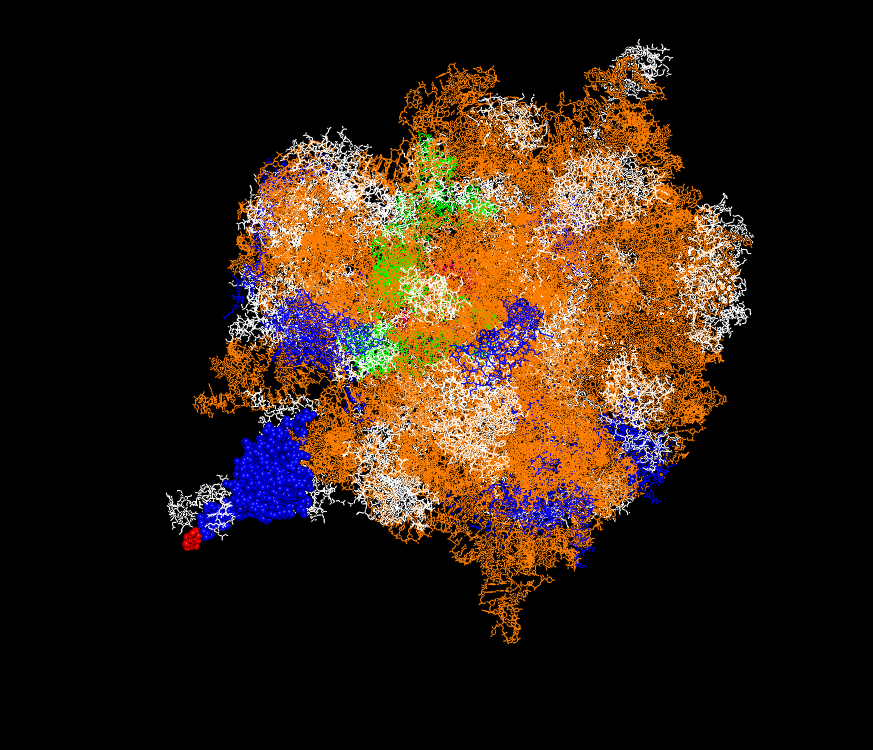 |
| --- |
| Figure 6. Crystal structure of the bacterial ribosomal L10 protein together with other ribosomal proteins and RNAs. |

| 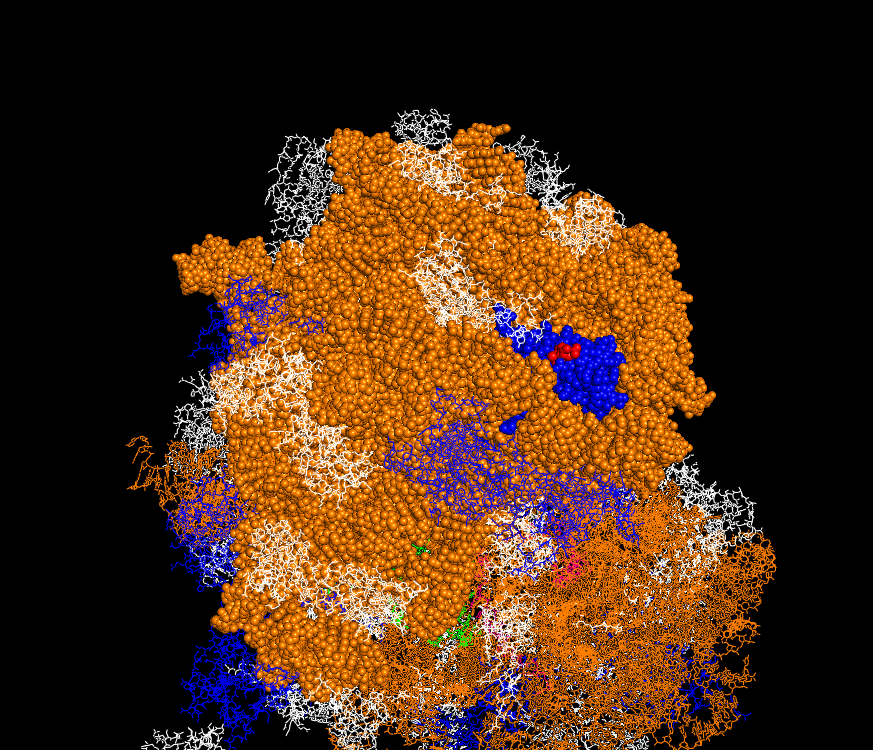 |
| --- |
| Figure 7. Crystal structure of the bacterial ribosomal L17 protein together with other ribosomal proteins and RNAs. |

| 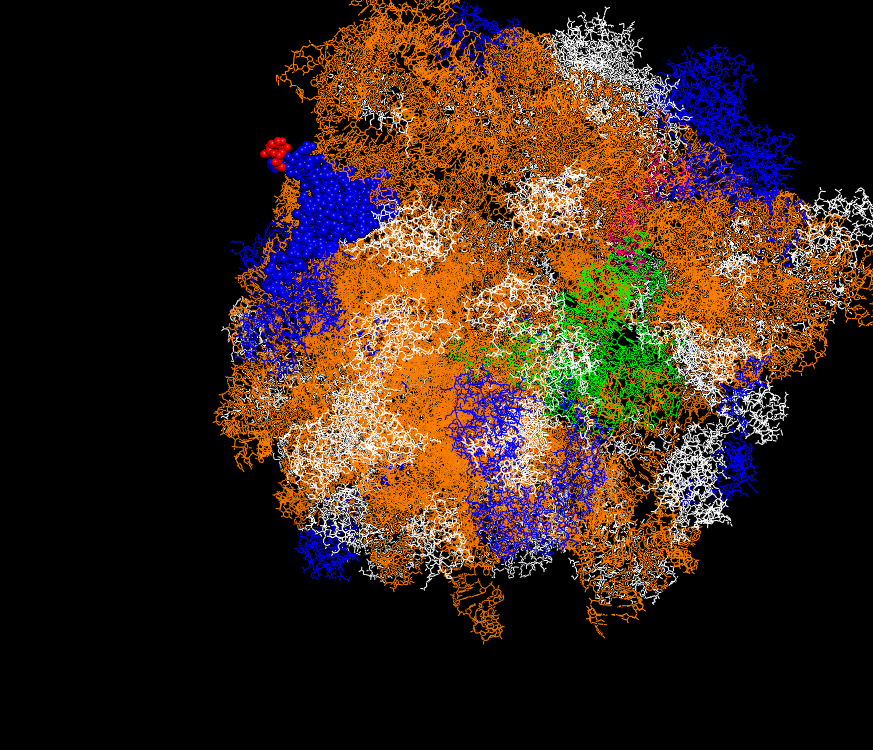 |
| --- |
| Figure 8. Crystal structure of the bacterial ribosomal L19 protein together with other ribosomal proteins and RNAs. |

| 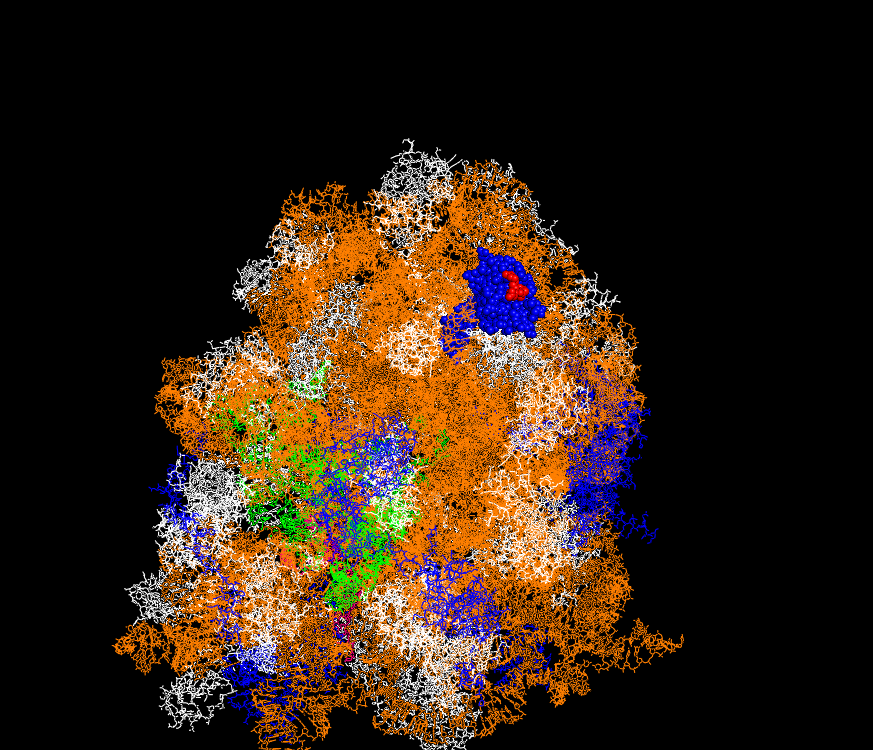 |
| --- |
| Figure 9. Crystal structure of the bacterial ribosomal L21 protein together with other ribosomal proteins and RNAs. |

| 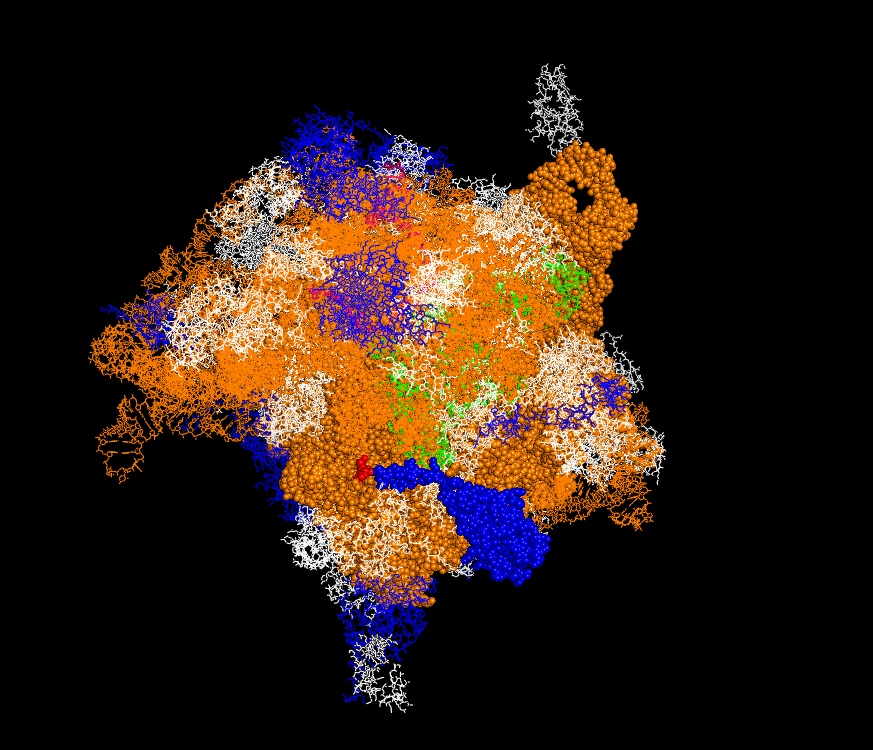 |
| --- |
| Figure 10. Crystal structure of the bacterial ribosomal L25 protein together with other ribosomal proteins and RNAs. |

| 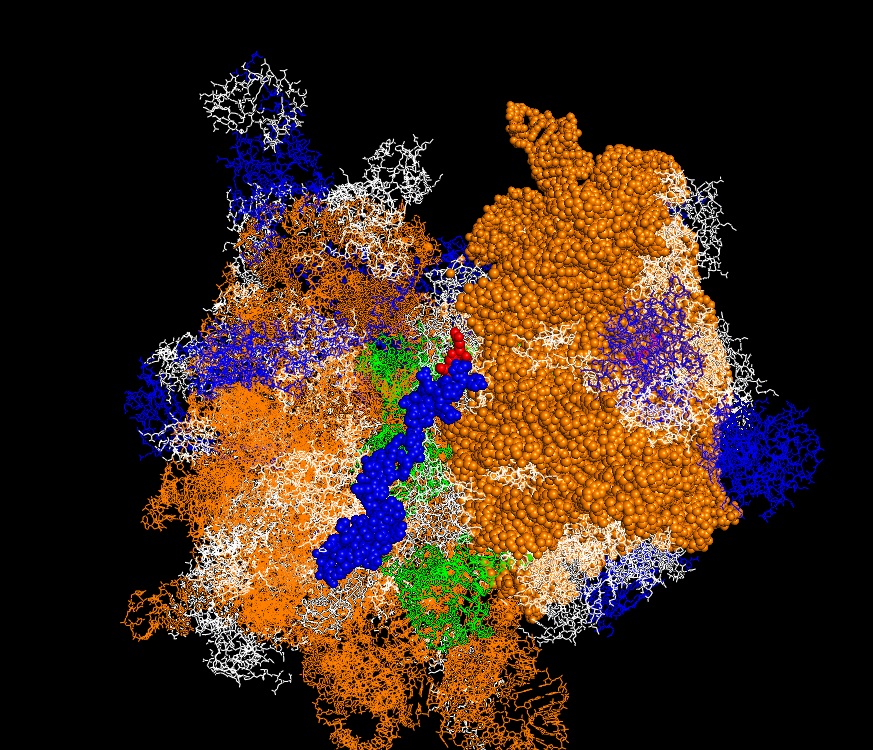 |
| --- |
| Figure 11. Crystal structure of the bacterial ribosomal L31 protein together with other ribosomal proteins and RNAs. |

| 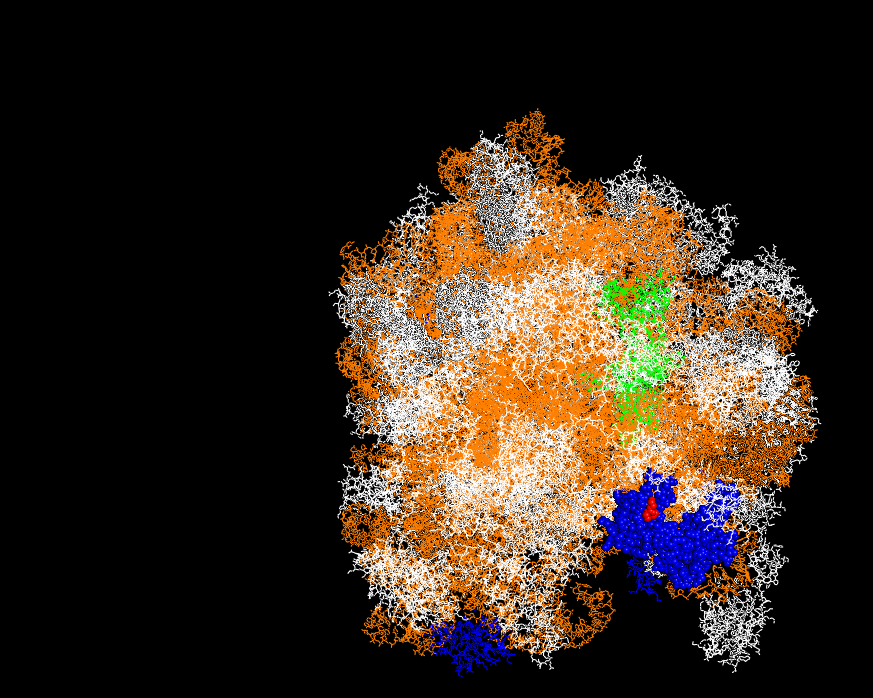 |
| --- |
| Figure 12. Crystal structure of the Archaeal ribosomal S3 protein together with other ribosomal proteins and RNAs. |

| 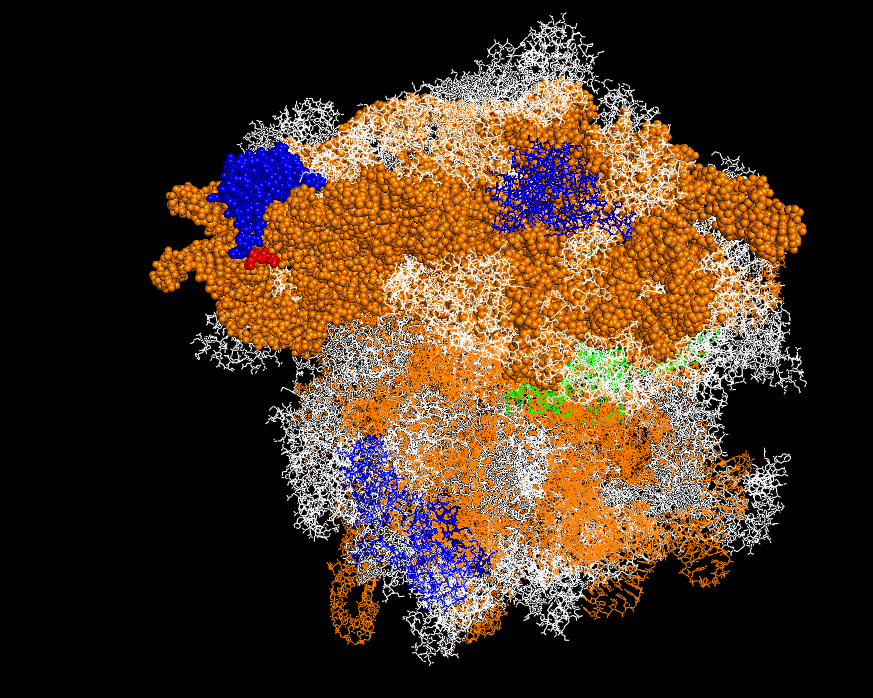 |
| --- |
| Figure 13. Crystal structure of the archaeal ribosomal S24 protein together with other ribosomal proteins and RNAs. |

| 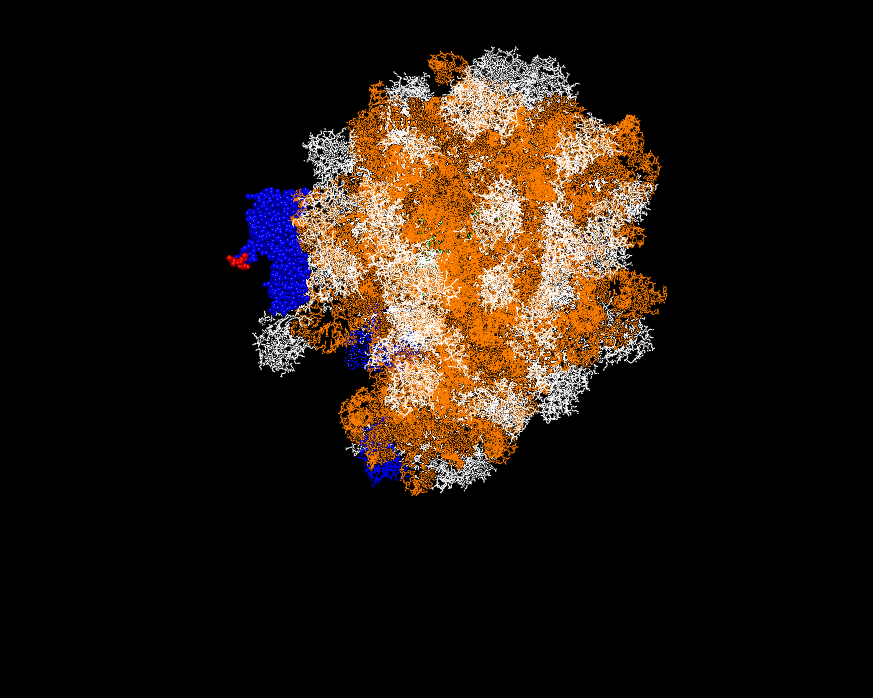 |
| --- |
| Figure 14. Crystal structure of the archaeal ribosomal L10 protein together with other ribosomal proteins and RNAs. |

| 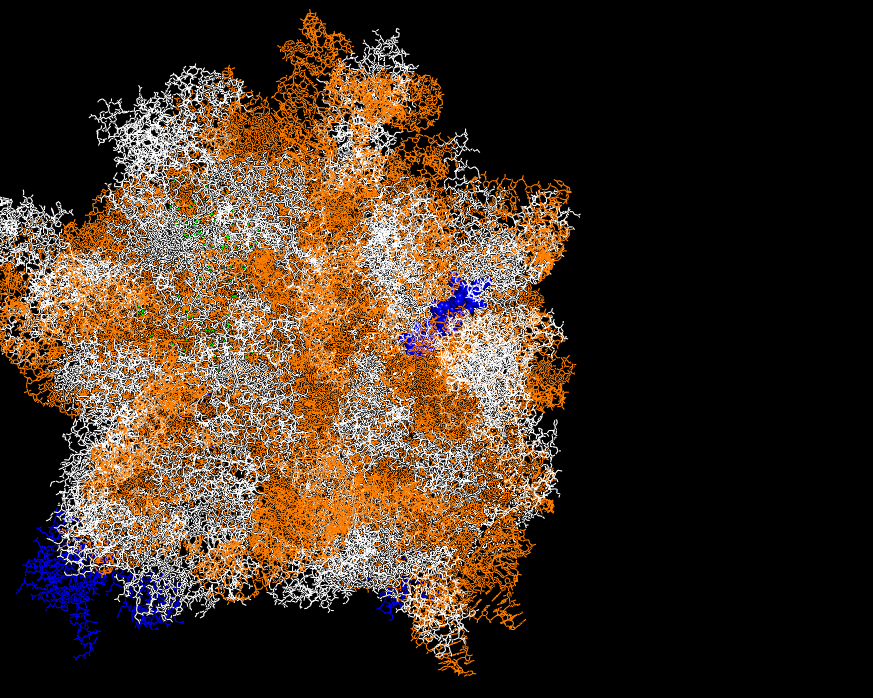 |
| --- |
| Figure 15. Crystal structure of the archaeal ribosomal L12 protein together with other ribosomal proteins and RNAs. |

|  |
| --- |
| Figure 16. The vast majority of ribosomal LCRs are located at the C-terminal part of their protein. |
